# Advances of native and non-native Antarctic species to *in vitro* conservation: improvement of disinfection protocols

**DOI:** 10.1038/s41598-020-60533-1

**Published:** 2020-03-02

**Authors:** Marely Cuba-Díaz, Claudia Rivera-Mora, Eduardo Navarrete, Macarena Klagges

**Affiliations:** 1 Laboratorio de Biotecnología y Estudios Ambientales, Universidad de Concepción, Campus Los Ángeles, Los Ángeles, Chile; 2 Departamento de Ciencias y Tecnología Vegetal, Escuela de Ciencias y Tecnologías, Universidad de Concepción, Campus Los Ángeles. Juan Antonio Coloma 0201, 4440000 Los Ángeles, Chile

**Keywords:** Agricultural genetics, Plant genetics

## Abstract

Plants that inhabit Antarctica have raised scientific interest due to their resilience to climate change, abiotic tolerance mechanisms and potential biological applications. *In vitro* propagation is useful for conservation, genetic material availability of these species and avoiding mass collection in their habitat. *In vitro* culture protocols for the native plants *Colobanthus quitensis* and *Deschampsia antarctica* and the non-native *Juncus bufonius* have been affected by endophytic microorganisms that proliferate when introduced to tissue cultures. This study evaluated the microbicidal and phytotoxic effect of calcium hypochlorite (Ca(ClO)_2_), silver nitrate (AgNO_3_) and silver nanoparticles (AgNPs), and their use at different concentrations for different time periods. The Ca(ClO)_2_ at 100 mg mL^−1^ showed the best microbial contamination control in *D. antarctica* (applied for 20 min) and for the three *C. quitensis* populations (applied for 15 min). In *J. bufonius*, AgNO_3_ at 10 mg mL^−1^ for 10 min reduced the microbial growth, but oxidative damage was generated. AgNPs did not prevent contamination or have adverse effects on tissues. Survival plantlets from each treatment, population or species were effectively introduced to the tissue culture and their propagation was successful. These results constitute a fundamental advance for the introduction, propagation and conservation of Antarctic species and their use in scientific research.

## Introduction

Global loss of biodiversity has increased considerably in recent years, which has made implementing conservation strategies essential for preserving and propagating plant genetic resources in each geographical region^1–3^. One method of conservation is through the use of germplasm banks, or facilities and centers created to conserve genetic resources under favorable conditions to prolong their survival; the final objective is the *ex-situ* conservation of specific genetic diversity. They are a source of material that enables many possibilities for a species of interest, from developing new cultivable varieties, biological technologies, or to establishing ecological restoration plans^4^.

The Antarctic continent is an extreme ecosystem due to its adverse environmental conditions. Its organisms are exposed to restricted availability of water and nutrients, very low temperatures, frequent freezing and thawing cycles, prolonged periods of darkness in the winter and exposure to high UV radiation levels during summer. These environmental characteristics are inhospitable to most organisms that live in temperate areas^5^. In addition, the increase in human activity in the region, and the confirmation that Antarctica is one of the critical points of global warming, make it a continent of highest priority for the conservation and development of multinational and multidisciplinary scientific challenges^6,7^.

There are only two native flowering plants that have been able to colonize some ice-free areas in the Antarctic Peninsula: *Deschampsia antarctica* Desv. (Poaceae) and *Colobanthus quitensis* (Kunth.) Bartl. (Caryophyllaceae)^8^. Some introduced species have also been reported, e.g. *Poa annua* L. (Poaceae)^9^ and *Juncus bufonius* L. var. bufonius (Juncaceae)^10,11^. Studies analyzing the mechanisms of adaptation, expansion and permanence of these species in the extreme conditions of the Antarctic ecosystem, as well as their potential biotechnological applications, have led to the development of conservation and propagation programs for these species in *ex situ* conditions. Several authors have reported advances in the development of tissue cultures for these species^12–15^, including studies that evidenced the differential responses to tissue cultures of different populations of *C. quitensis*^16,17^. However, the *in vitro* establishment of these species has been hampered due to endophytic microorganisms^18–22^ that develop in the nutritive media once the explants are placed in them. This causes direct and indirect tissue mortality, necrosis, reduced proliferation of roots and shoots and, in some cases, the death of tissues or plants in their entirety^23^. This microbial contamination has been recognized as the most serious limiting factor for research on vegetable tissue cultures and commercial applications^24–26^. The disinfection of plant material is the first step for the establishment of a crop in *in vitro* conditions, which consists of eliminating contaminants such as exogenous and endogenous bacteria and fungi without damaging the plant tissue, thus ensuring the success of the culture^27^. To avoid contamination, the following are commonly used: antibiotics, fungicides, sodium hypochlorite (NaClO), ethanol, hydrogen peroxide (H_2_O_2_), mercury bichloride (HgCl_2_), calcium hypochlorite (Ca(ClO)_2_) and silver nitrate (AgNO_3_)^28^. Silver nanoparticles (AgNPs) have also been used more recently due to their microbicidal properties at low concentrations^29^.

In previous protocols for introducing Antarctic native species to *in vitro* cultures carried out by Cuba *et al*.^12^ and Zúñiga *et al*.^13^, NaClO was used as a disinfectant at different concentrations, but it has not been possible to avoid microorganism proliferation in the cultures in an optimal form. Fungicides and HgCl_2_ have also been evaluated; however, they have not been able to prevent microorganism development either, and HgCl_2_ has caused explant death. Thus, the goal of this study is to evaluate the microbicidal and phytotoxic effects of calcium hypochlorite (Ca(ClO)_2_), silver nitrate (AgNO_3_) and silver nanoparticles (AgNPs) disinfectant agents in the *in vitro* establishments of the species *C. quitensis*, *D. antarctica* and *J. bufonius* in order to reduce the undesired effects of endophytic microorganisms in the process.

## Materials and Methods

### Plant material

For *C. quitensis* species, plants from three populations were used: (i) Arctowski-pA (King George Island, Antarctic, 62 °09’S, 58 °28’W, 3–23 m a.s.l.), (ii) Conguillío-pC (Conguillío National Park, Chile, 38 °36’S, 71 °36’W, 2575 m a.s.l.) and (iii) Laredo-pL (north of Punta Arenas, Chile, 52 °09’S, 70 °40’W, 158 m a.s.l). These plants, as well as *D. antarctica* and *J. bufonius* (both from the same site as Arctowski *C. quitensis* populations) were collected from their natural habitats and maintained in growth chambers in 240 cc polystyrene containers in soil: peat: pearlite (3:2:1) at 14 ± 1 °C, for a photoperiod of 16/8 hours light/dark, with a photonic flux of 100 ± 20 µmol m^−2^ s^−1^ photons and relative humidity of 75 ± 5% with manual irrigation at field capacity (approximately 30 ml). The plants were fertilized every 2 weeks with 0.2 g L^−1^ of complete Phostrogen fertilizer (NPK 13:10:27).

### Culture medium

Explants from all species and populations were planted in test tubes containing 2.5 ml of culture medium. Murashige & Skoog salts and vitamins (MS) (PhytoTechnology Laboratories®) supplemented with sucrose (3%), 0.5 mg L^−1^ of 6-benzylaminopurine (BAP), with 7 g L^−1^ of Agar (National Agar Agar Soviquim Ltda) added as gelling agent. For *C. quitensis* populations, 0.25 mg L^−1^ of indole acetic acid (IAA) and 10 µM of Silver thiosulphate (STS) were also added. The culture medium was adjusted at 5.8 pH and sterilized at 120 °C at 115 kPa for 20 min. STS was added for *C*. *quitensis*^15^ under a laminar flow chamber once the culture medium reached approximately 30 °C. All culture media were dispensed in sterile test tubes at a volume of 2.5 mL.

### Disinfection solutions

The solutions of AgNO_3_(Soviquim Ltda.), Ca(ClO)_2_ (Winkler Ltda.), AgNPs (Nano Tec SA, Chile) and Clorox® (4.9% NaClO) were prepared under a laminar flow chamber with sterile distilled water. The Ca(ClO)_2_ solution was stirred for 4 hours, left to stand overnight and filtered under a vacuum in aseptic conditions. The AgNPs of 50 nm size used were dissolves in ethanol, where the initial additive contained 80% Ag and 20% ethanol, and their morphology was spherical amorphous. The fungicide (Tebuconazol 2% + Carbendazima 1%; Anasac) was prepared using distilled water outside the laminar flow chamber.

### Sterilization treatment

The explants were washed in water to eliminate the soil present in the root system. Cuts were made in the roots and leaves to favor the penetration of the disinfectant into the tissue. They were subsequently placed in 800 mL of tap water with 3 drops of commercial dishwashing liquid (Quix) in agitation for 5 min and rinsed repeatedly with running water. They were disinfected with fungicide (Tebuconazol 2%+Carbendazima 1%) at 2.5 mg mL^−1^ for 30 min in agitation, and rinsed 3 more times with sterile distilled water. The explants were left in distilled water to avoid tissue dehydration. In the laminar flow chamber, the disinfection treatments containing Ca(ClO)_2_, AgNO_3_ and AgNPs were applied with the concentrations and time periods described in Table 1^28–33^.

**Table 1:** Disinfection treatments applied to the explants of *Colobanthus quitensis*, *Deschampsia antarctica* and *Juncus bufonius*.

Treatment	Disinfectant	Time
Control	Sodium hypochlorite 4.9 mg mL^−1^	10 minutes (*C. quitensis*)
	Sodium hypochlorite 7.4 mg mL^−1^	20 minutes (*D. antarctica* and *J. bufonius*)
T1	Calcium hypochlorite 50 mg mL^−1^	15 minutes
T2	Calcium hypochlorite 50 mg mL^−1^	20 minutes
T3	Calcium hypochlorite 100 mg mL^−1^	15 minutes
T4	Calcium hypochlorite 100 mg mL^−1^	20 minutes
T5	Silver nitrate 5 mg mL^−1^	5 minutes
T6	Silver nitrate 5 mg mL^−1^	10 minutes
T7	Silver nitrate 10 mg mL^−1^	5 minutes
T8	Silver nitrate 10 mg mL^−1^	10 minutes
T9	Silver nanoparticles 0.1 mg mL^−1^	60 minutes
T10	Silver nanoparticles 0.1 mg mL^−1^	90 minutes
T11	Silver nanoparticles 0.2 mg mL^−1^	60 minutes
T12	Silver nanoparticles 0.2 mg mL^−1^	90 minutes

As a control treatment, the methodology from the Biotechnology and Environmental Studies Laboratory (Laboratorio de Biotecnología y Estudios Ambientales, Los Ángeles, Chile) was applied: NaClO at 4.9 mg mL^−1^ for 10 min for *C. quitensis;* NaClO at 7.4 mg mL^−1^ for 20 min for *D. antarctica* and *J. bufonius*. The plants were then washed 3 times for 1 min with 200 mL of sterile distilled water and dried with sterile absorbent paper. Stem explants were prepared by cutting the rest of the roots and leaves. Fragments of 5 mm in length were obtained and placed in the specific culture medium for each species. One explant was left in each test tube in a growth chamber at 20 ± 2 °C with a photoperiod of 16/8 hours light/dark, with a light intensity of 50 ± 5 µmol m^−2^ s^−1^ photons.

After 4 weeks post-treatment, all explants without contamination and that evidenced tissue prolifaration were subcultured to culture media with the same composition. After 2 months, qualitative observations were made regarding root proliferation, the appearance of flowers or flower apices, and the presence of yellowness or necrosis symptoms.

### Data collection

To evaluate the phytotoxic effect of the disinfectants, the number of explants that showed oxidation and the number of dead explants were recorded after 1 week. Then, 4 weeks after disinfection, the number of explants that showed contamination, the number of plants that developed shoots, roots and flowers, and the number of these structures per plantlet were recorded. With this data, the percentage of plantlets that showed shoots, roots and flowers, and the percentages of contamination and survival were calculated.

All experiments included completely randomized designs with n = 15, where 5 explants were used per treatment in 3 repetitions. For the statistical analysis, the data expressed in percentages were transformed by applying arcsine √%/100. To determine significant differences between treatments (p < 0.05), variables that fulfilled the assumptions of normality, homogeneity of variances and independence were analyzed via Duncan’s Multiple Range Test, and variables that did not have a normal distribution or did not present homogeneity of variances were analyzed via Dunn’s nonparametric test (the variables are specified in Tables 2–6).

**Table 2:** Percentage of contamination, plantlet survival, plantlet oxidation and shoot proliferation, and shoot number per plantlet in plantlets of the Conguillío population of *Colobanthus quitensis* after 4 weeks post-disinfection treatments.

Conguillío						
Treatment		Contamination (%)*	Survival (%)*	Oxidation (%)*	Shoots (%)*	Shoots number*
Control	NaClO at 4.9 mg mL^−1^ for 10 min.	100 ± 0a	100 ± 0a	0 ± 0b	0 ± 0a	0 ± 0a
T1	Ca(ClO)_2_ at 50 mg mL^−1^ for 15 min.	86.67 ± 11.55ab	100 ± 0a	0 ± 0b	13.33 ± 11.55a	0.60 ± 0.6a
T2	Ca(ClO)_2_ at 50 mg mL^−1^ for 20 min.	93.33 ±11.55ab	100 ± 0a	0 ± 0b	6.67 ± 11.55a	0.27 ± 0.46a
T3	Ca(ClO)_2_ at 100 mg mL^−1^ for 15 min.	80 ± 0b	100 ± 0a	0 ± 0b	13.33 ± 11.55a	0.67 ± 0.12a
T4	Ca(ClO)_2_ at 100 mg mL^−1^ for 20 min.	100 ± 0a	100 ± 0a	0 ± 0b	0 ± 0a	0 ± 0a
T5	AgNO3 at 5 mg mL^−1^ for 5 min.	80 ± 0b	100 ± 0a	100 ± 0a	6.67 ± 11.55a	0.07 ± 0.12a
T6	AgNO3 at 5 mg mL^−1^ for 10 min.	86.67 ± 11.55ab	100 ± 0a	100 ± 0a	6.67 ± 11.55a	0.33 ± 0.42a
T7	AgNO3 at 10 mg mL^−1^ for 5 min.	86.67 ± 11.55ab	100 ± 0a	100 ± 0a	6.67 ± 11.55a	0.27 ± 0.46a
T8	AgNO3 at 10 mg mL^−1^ for 10 min.	80 ± 34.64ab	100 ± 0a	100 ± 0a	20 ± 0a	0.47 ± 0.50a
T9	AgNPs at 0.1 mg mL^−1^ for 60 min.	100 ± 0a	100 ± 0a	0 ± 0b	0 ± 0a	0 ± 0a
T10	AgNPs at 0.1 mg mL^−1^ for 90 min.	100 ± 0a	100 ± 0a	0 ± 0b	0 ± 0a	0 ± 0a
T11	AgNPs at 0.2 mg mL^−1^ for 60 min.	100 ± 0a	100 ± 0a	0 ± 0b	0 ± 0a	0 ± 0a
T12	AgNPs at 0.2 mg mL^−1^ for 90 min.	100 ± 0a	100 ± 0a	0 ± 0b	0 ± 0a	0 ± 0a

Mean ± standard deviation. *Dunn’s Test. Equal letters did not differ statistically (p < 0.05).

**Table 3:** Percentage of contamination, plantlet survival, plantlet oxidation and proliferation of shoots and flowers, and numbers of shoots and flowers per plantlet in plantlets of the Arctowski population of *Colobanthus quitensis* after 4 weeks post-disinfection treatments.

Arctowski								
Treatment		Contamination (%)**	Survival (%)*	Oxidation (%)*	Shoots (%)**	Shoots number*	Flowers (%)**	Flowers number*
Control	NaClO at 4.9 mg mL^−1^ for 10 min.	100 ± 0a	100 ± 0a	0 ± 0b	0 ± 0e	0 ± 0b	0 ± 0b	0 ± 0a
T1	Ca(ClO)_2_ at 50 mg mL^−1^ for 15 min.	26.66 ± 11.54b	100 ± 0a	0 ± 0b	60 ± 0abc	1.44 ± 0.53a	20 ± 20ab	0.4 ± 0.4a
T2	Ca(ClO)_2_ at 50 mg mL^−1^ for 20 min.	13.33 ± 23.09b	100 ± 0a	0 ± 0b	73.33 ± 30.55ab	2 ± 0.72a	33.33 ± 30.55ab	0.33 ± 0.31a
T3	Ca(ClO)_2_ at 100 mg mL^−1^ for 15 min.	13.33 ± 11.54b	86.66 ± 11.54a	0 ± 0b	73.33 ± 23.09ab	2 ± 0.53a	6.67 ± 11.55ab	0.07 ± 0.12a
T4	Ca(ClO)_2_ at 100 mg mL^−1^ for 20 min.	13.33 ± 11.54b	100 ± 0a	0 ± 0b	93.33 ± 11.55a	2.8 ± 1.4a	26.67 ± 30.55ab	0.33 ± 0.31a
T5	AgNO3 at 5 mg mL^−1^ for 5 min.	20 ± 20b	100 ± 0a	100 ± 0a	73.33 ± 30.55ab	1.53 ± 0.23a	26.67 ± 30.55ab	0.47 ± 0.23a
T6	AgNO3 at 5 mg mL^−1^ for 10 min.	20 ± 0b	93.33 ± 11.54a	100 ± 0a	46.67 ± 30.55bcd	0.93 ± 0.31ab	40 ± 20ab	0.6 ± 0.2a
T7	AgNO3 at 10 mg mL^−1^ for 5 min.	33.33 ± 23.09b	93.33 ± 11.54a	100 ± 0a	53.33 ± 30.55bcd	0.93 ± 0.31ab	26.67 ± 30.55ab	0.4 ± 0.53a
T8	AgNO3 at 10 mg mL^−1^ for 10 min.	20 ± 0b	100 ± 0a	100 ± 0a	66.67 ± 11.55abc	1.47 ± 0.31a	46.67 ± 30.55a	0.8 ± 0.87a
T9	AgNPs at 0.1 mg mL^−1^ for 60 min.	86.66 ± 23.09a	100 ± 0a	100 ± 0a	26.67 ± 23.09cde	0.2 ± 0.35b	26.67 ± 30.55ab	0.4 ± 0.53a
T10	AgNPs at 0.1 mg mL^−1^ for 90 min.	86.66 ± 11.54a	100 ± 0a	100 ± 0a	13.33 ± 11.55de	0.2 ± 0.2b	13.33 ± 11.55ab	0.2 ± 0.2a
T11	AgNPs at 0.2 mg mL^−1^ for 60 min.	86.66 ± 11.54a	100 ± 0a	100 ± 0a	33.33 ± 23.09bcd	0.73 ± 0.42ab	0 ± 0b	0 ± 0a
T12	AgNPs at 0.2 mg mL^−1^ for 90 min.	86.66 ± 23.09a	100 ± 0a	100 ± 0a	0 ± 0e	0 ± 0b	13.33 ± 23.09ab	0.27 ± 0.46a

Mean ± standard deviation. *Dunn’s Test, **Duncan’s Test. Equal letters did not differ statistically (p < 0.05).

**Table 4:** Percentage of contamination, plantlet survival, plantlet oxidation and proliferation of shoots and roots and numbers of shoots and roots per plantlet in plantlets of the Laredo population of *Colobanthus quitensis* after 4 weeks post-disinfection treatments.

Laredo								
Treatment		Contamination(%)*	Survival (%)**	Oxidation (%)**	Shoots (%)*	Shoots number*	Roots (%)*	Roots number*
Control	NaClO at 4.9 mg mL^−1^ for 10 min.	100 ± 0a	100 ± 0a	0 ± 0b	0 ± 0b	0 ± 0b	0 ± 0a	0 ± 0a
T1	Ca(ClO)_2_ at 50 mg mL^−1^ for 15 min.	20 ± 0b	93.33 ± 11.55a	0 ± 0b	60 ± 20a	1 ± 0a	6.67 ± 11.55a	0 ± 0a
T2	Ca(ClO)_2_ at 50 mg mL^−1^ for 20 min.	33.33 ± 11.55b	86.67 ± 23.09a	0 ± 0b	20 ± 20ab	0 ± 0ab	6.67 ± 11.55a	0 ± 0a
T3	Ca(ClO)_2_ at 100 mg mL^−1^ for 15 min.	33.33 ± 23.09b	100 ± 0a	0 ± 0b	53.33 ± 30.55a	1 ± 1a	26.67 ± 23.09a	1 ± 1a
T4	Ca(ClO)_2_ at 100 mg mL^−1^ for 20 min.	13.33 ± 23.09b	93.33 ± 11.55a	0 ± 0b	33.33 ± 41.63ab	1 ± 1ab	30 ± 42.43a	1 ± 1a
T5	AgNO3 at 5 mg mL^−1^ for 5 min.	53.33 ± 41.63ab	86.67 ± 11.55a	100 ± 0a	26.67 ± 23.09ab	0 ± 0ab	0 ± 0a	0 ± 0a
T6	AgNO3 at 5 mg mL^−1^ for 10 min.	86.67 ± 11.55ab	86.67 ± 11.55a	100 ± 0a	0 ± 0b	0 ± 0b	0 ± 0a	0 ± 0a
T7	AgNO3 at 10 mg mL^−1^ for 5 min.	73.33 ± 11.55ab	100 ± 0a	100 ± 0a	13.33 ± 23.09ab	0 ± 0b	0 ± 0a	0 ± 0a
T8	AgNO3 at 10 mg mL^−1^ for 10 min.	40 ± 40b	73.33 ± 30.55a	100 ± 0a	20 ± 20ab	1 ± 0ab	0 ± 0a	0 ± 0a
T9	AgNPs at 0.1 mg mL^−1^ for 60 min.	86.67 ± 11.55a	93.33 ± 11.55a	0 ± 0b	0 ± 0b	0 ± 0b	0 ± 0a	0 ± 0a
T10	AgNPs at 0.1 mg mL^−1^ for 90 min.	80 ± 20a	100 ± 0a	0 ± 0b	20 ± 20ab	0 ± 0ab	0 ± 0a	0 ± 0a
T11	AgNPs at 0.2 mg mL^−1^ for 60 min.	100 ± 0a	86.67 ±23.09a	0 ± 0b	0 ± 0b	0 ± 0b	0 ± 0a	0 ± 0a
T12	AgNPs at 0.2 mg mL^−1^ for 90 min.	100 ± 0a	86.67 ± 11.55a	0 ± 0b	0 ± 0b	0 ± 0b	0 ± 0a	0 ± 0a

Mean ± standard deviation. *Dunn’s Test, **Duncan’s Test. Equal letters did not differ statistically (p < 0.05).

**Table 5:** Percentage of contamination, plantlet survival, plantlet oxidation and proliferation of shoots and roots and numbers of shoots, leaves and roots per plantlet in plantlets of *Deschampsia antarctica* after 4 weeks post-disinfection treatments.

*Deschampsia antarctica*									
Treatment		Contamination (%)**	Survival (%)**	Oxidation (%)*	Shoots (%)*	Shoots number*	Leaves number*	Roots (%)*	Roots number*
Control	NaClO at 7.4 mg mL^−1^ for 20 min.	73.33 ± 11.55ab	93.33 ± 11.55ab	0 ± 0b	13.33 ± 23.09a	0.13 ± 0.23a	1.47 ± 1.15a	20 ± 20a	1.2 ± 1a
T1	Ca(ClO)_2_ at 50 mg mL^−1^ for 15 min.	66.67 ± 34.64ab	86.67 ± 11.55abc	0 ± 0b	20 ± 20a	0.47 ± 0.50a	4 ± 5.44a	13.33 ± 23.09a	0.73 ± 1a
T2	Ca(ClO)_2_ at 50 mg mL^−1^ for 20 min.	40 ± 34.64bc	73.33 ± 11.55bcd	0 ± 0b	13.33 ± 11.55a	0.07 ± 0.12a	2.4 ± 2.16a	20 ± 20a	0.4 ± 0a
T3	Ca(ClO)_2_ at 100 mg mL^−1^ for 15 min.	20 ± 20c	60 ± 20cd	0 ± 0b	26.67 ± 23.09a	0.33 ± 0.31a	3.27 ± 1.86a	26.67 ± 23.09a	0.67 ± 1a
T4	Ca(ClO)_2_ at 100 mg mL^−1^ for 20 min.	33.33 ± 11.55bc	73.33 ± 11.55bcd	0 ± 0b	33.33 ± 11.55a	0.6 ± 0.35a	3.8 ± 1.59a	26.67 ± 11.55a	0.8 ± 0a
T5	AgNO3 at 5 mg mL^−1^ for 5 min.	73.33 ± 11.55ab	86.67 ± 11.55abc	100 ± 0a	13.33 ± 11.55a	0.4 ± 0.4a	2 ± 1.74a	13.33 ± 11.55a	0.6 ± 1a
T6	AgNO3 at 5 mg mL^−1^ for 10 min.	46.67 ± 30.55bc	66.67 ± 11.55cd	100 ± 0a	20 ± 11.55a	0.2 ± 0.2a	1.73 ± 1.62a	20 ± 20a	0.47 ± 1a
T7	AgNO3 at 10 mg mL^−1^ for 5 min.	33.33 ± 30.55bc	80 ± 20abcd	100 ± 0a	40 ± 20a	0.7 ± 0.64a	5.27 ± 5.16a	33.33 ± 23.09a	0.6 ± 1a
T8	AgNO3 at 10 mg mL^−1^ for 10 min.	26.67 ± 30.55bc	53.33 ± 30.55d	100 ± 0a	26.67 ± 30.55a	0.4 ± 0.53a	4.2 ± 6.43a	33.33 ± 30.55a	0.67 ± 1a
T9	AgNPs at 0.1 mg mL^−1^ for 60 min.	93.33 ± 11.55a	100 ± 0a	0 ± 0b	0 ± 0a	0 ± 0a	0 ± 0a	26.67 ± 0a	0.2 ± 0a
T10	AgNPs at 0.1 mg mL^−1^ for 90 min.	100 ± 0a	100 ± 0a	0 ± 0b	0 ± 0a	0 ± 0a	0 ± 0a	26.67 ± 0a	0 ± 0a
T11	AgNPs at 0.2 mg mL^−1^ for 60 min.	100 ± 0a	100 ± 0a	0 ± 0b	0 ± 0a	0 ± 0a	0 ± 0a	20 ± 0a	0 ± 0a
T12	AgNPs at 0.2 mg mL^−1^ for 90 min.	93.33 ± 11.55a	80 ± 20abcd	0 ± 0b	0 ± 0a	0 ± 0a	0 ± 0a	26.67 ± 0a	0 ± 0a

Mean ± standard deviation. *Dunn’s Test, **Duncan’s Test. Equal letters did not differ statistically (p < 0.05).

**Table 6:** Percentage of contamination, plantlet survival, plantlet oxidation and proliferation of shoots, roots and flowers (inflorescence), and numbers of shoots, leaves, roots and flowers per plantlet in plantlets of *Juncus bufonius* after 4 weeks post-disinfection treatments.

*Juncus bufonius*											
Treatment		Contamination (%)**	Survival (%)**	Oxidation (%)**	Shoots (%)**	Shoots Number*	Leaves number*	Roots (%)*	Roots number*	Flowers (%)*	Flowers number*
Control	NaClO at 7.4 mg mL^−1^ for 20 min.	40 ± 0ab	80 ± 0a	66.67 ± 11.55a	53.33 ± 11.55a	1.47 ± 1a	9.20 ± 5.44a	53.33 ± 11.55a	1.13 ± 1.27a	13.33 ± 23.09a	0.2 ± 0.35a
T1	Ca(ClO)_2_ at 50 mg mL^−1^ for 15 min.	26.67 ± 11.55ab	53.33 ± 11.55ab	73.33 ± 11.55a	26.67 ± 11.55ab	1.07 ± 1b	4.20 ± 2.62ab	6.67 ± 11.55a	0.07 ± 0.12a	6.67 ± 11.55a	0.07 ± 0.12a
T2	Ca(ClO)_2_ at 50 mg mL^−1^ for 20 min.	33.33 ± 11.55ab	55.67 ± 5.77ab	60 ± 0a	13.33 ± 23.09ab	0.13 ± 0ab	2.93 ± 2.05ab	13.33 ± 23.09a	0.47 ± 0.6a	6.67 ± 11.55a	0.33 ± 0.58a
T3	Ca(ClO)_2_ at 100 mg mL^−1^ for 15 min.	26.67 ± 30.55ab	66.67 ± 11.55ab	73.33 ± 30.55a	20 ± 34.64ab	0.6 ± 1ab	3.53 ± 6.12ab	13.33 ± 23.09a	0.27 ± 0.46a	0 ± 0a	0 ± 0a
T4	Ca(ClO)_2_ at 100 mg mL^−1^ for 20 min.	26.67 ± 23.09ab	66.67 ± 23.09ab	80 ± 20a	0 ± 0b	0 ± 0b	0 ± 0b	0 ± 0a	0 ± 0a	6.67 ± 11.55a	0.07 ± 0.12a
T5	AgNO3 at 5 mg mL^−1^ for 5 min.	13.03 ± 23.09ab	66.67 ± 23.09ab	86.67 ± 23.09a	33.33 ± 11.55a	0.6 ± 1ab	6.87 ± 3.58ab	20 ± 20a	0.87 ± 1.17a	13.33 ± 23.09a	0.4 ± 0.69a
T6	AgNO3 at 5 mg mL^−1^ for 10 min.	20 ± 20ab	60 ±0ab	86.67 ± 23.09a	33.33 ± 30.55ab	0.53 ± 1ab	3.80 ± 3.47ab	6.67 ± 11.55a	0.07 ± 0.12a	13.33 ± 23.09a	0.2 ± 0.35a
T7	AgNO3 at 10 mg mL^−1^ for 5 min.	46.67 ± 11.55a	66.67 ± 30.55a	53.33 ± 11.55a	40 ± 20a	0.6 ± 0ab	4.47 ± 2.12ab	20 ± 20a	0.27 ± 0.23a	6.67 ± 11.55a	0.13 ± 0.23a
T8	AgNO3 at 10 mg mL^−1^ for 10 min.	6.67 ± 11.55b	33.33 ± 30.55b	86.67 ± 11.55a	13.33 ± 23.09ab	0.4 ± 1ab	2.33 ± 2.21ab	6.67 ± 11.5a	0.07 ± 0.12a	0 ± 0a	0 ± 0a

Mean ± standard deviation. *Dunn’s Test, **Duncan’s Test. Equal letters did not differ statistically (p < 0.05).

## Results

**Figure 1:**
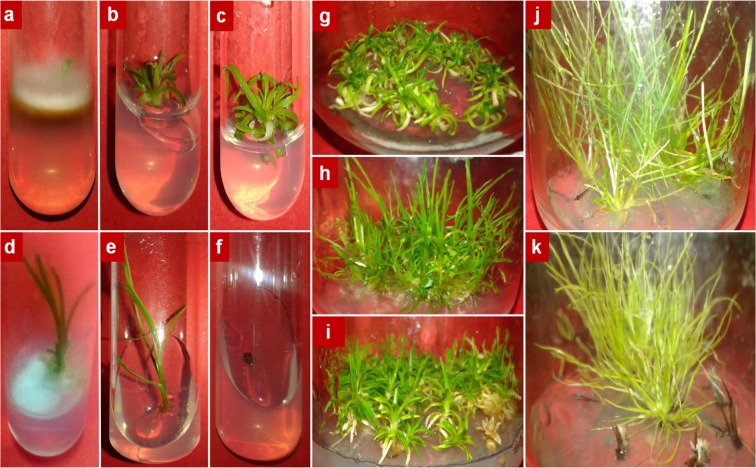
*Colobanthus quitensis* explants after the disinfection treatments. (**a**) Arctowski: AgNPs 0.2 mg mL^−1^ for 60 min after one week and (**b**) 100 mg mL^−1^ Ca(ClO)_2_ for 15 min after 4 weeks; (**c**) Laredo: 100 mg mL^−1^ Ca(ClO)_2_ for 15 min after 4 weeks; (**d**) Conguillío: AgNO_3_ 5 mg mL^−1^ for 5 min after one week; (**e**) *Deschampsia antarctica:* 100 mg mL^−1^ Ca(ClO)_2_ for 20 min after 4 weeks and (**f**) *Juncus bufonius:* 10 mg mL^−1^ AgNO_3_ for 10 min at 4 weeks. Plantlets after 2 months of subculture: (**g**) Laredo; (**h**) Conguillío; (**i**) Arctowski; (**j**) *D. antarctica* and (**k**) *J. bufonius*.

Responses to different disinfection treatments varied among the different populations of *C. quitensis*. In most treatments, microbial growth occurred during the first week after disinfection treatment. For the 3 populations of *C. quitensis*, the highest contamination was mainly fungal (Fig. 1a,d), averaging 93.1% among all disinfection treatment. While bacterial contamination only reached a maximum of 16% for Arctowski (pA) and Laredo (pL) populations disinfected with AgNO_3_ but was absent in most other treatments. The Conguillío population (pC) showed between 80–100% of contamination, the highest contamination percentages observed (Table 2), of which, on average 95% corresponded to fungal contamination. The Ca(ClO)_2_ at 100 mg mL^−1^ solution applied for different time periods was the treatment that showed the best results when analyzing the variables in this study. Thus, for pC, the 15 min treatment in Ca(ClO)_2_ solution, regardless of the concentration (50 or 100 mg mL^−1^), managed to control contamination by 20%. It did not affect explant survival and induced the appearance of shoots by about 13% (Table 2). In pC explants, only shoots were present and roots were absent. This was overcome when the shoots were subcultured to a fresh medium, where the development of new plantlets was successful (Fig. 1h). For both, pA and pL populations, the application of Ca(ClO)_2_ at 100 mg mL^−1^ for 15 or 20 min showed no significant differences (Tables 3, 4). When analyzing each evaluated variable, in pA, the treatment with 100 mg mL^−1^ of Ca(ClO)_2_ for 20 min led to a 100% survival of the explants and high shoot proliferation but also induced high flower proliferation. This variable showed lower values (20% lower) in the treatment for 15 min (Table 3, Fig. 1b). In pL the treatment at 100 mg mL^−1^ of Ca(ClO)_2_ for 15 min, despite allowing the appearance of contaminants in 20% more than in the treatment for 20 min, favored 100% survival of the explants and showed higher shoot proliferation (Table 4, Fig. 1c).

The AgNO_3_ treatment was not effective under the experimental conditions evaluated because although it allowed controlling microbial contamination, it produced 100% oxidation in the explants for the 3 *C. quitensis* populations studied (Tables 2–4). Even in pA, where this disinfectant agent was able to control the appearance of contamination up to 80%, symptoms of oxidation were produced within a few hours, and some plantlets experienced a slow growth compared to the other treatments. The damage increased as concentration and time of exposure increased (Table 3).

The AgNPs were not able to control contamination in any of the treatments applied to pC (Table 2). In pA, although the disinfectant was able to control about 14% of the contamination, it caused the oxidation of 100% of the tissues (Table 3). The higher concentrations of the agent also failed to prevent the appearance of contaminants in pL, and the lower concentrations did not facilitate tissue proliferation (Table 4).

In general, the *D. antarctica* species showed a similar behavior to that observed for *C. quitensis* in relation to the control of explant contamination and the predominance of fungal contamination (98%), compared to bacterial (15%) among each treatment applied. The Ca(ClO)_2_ at 100 mg mL^−1^ treatments showed the lowest percentages of contamination, 20% and 33.3% for 15 and 20 min (T3 and T4), respectively (Table 5). Although these two treatments did not show significant differences, the treatment for 20 min showed 13% explant survival and 6.6% higher shoot proliferation than the treatment performed for 15 min (Table 5, Fig. 1e). The AgNPs were not able to control contamination, and the application of AgNO_3_ at different concentrations and times caused oxidation in all explants within a few hours (Table 5). In all treatments, regardless of the oxidation of explants (AgNO_3_), or non-proliferation of shoots (AgNPs), root development occurred (Table 5).

In *J. bufonius*, contamination fluctuated between 6% and 46% (where approximately 75.7% of observed contamination was fungal), and was lower after the use of AgNO_3_ at 10 mg mL^−1^ solution for 10 min (T8). In all the treatments, the survival rate was less than 80% and more than 50% of explants showed oxidation. The T8 treatment had the lowest survival rate at 33% (Fig. 1f). After using Ca(ClO)_2_ at 100 mg mL^−1^ for 20 min (T4), there was no formation of shoots or roots, and only the development of inflorescences was observed. Shoots, roots and flowers were found for the rest of the treatments (Table 6). In most treatments, exudates of some oxidizing compound could be observed in the culture medium. Only the control treatment (NaClO 7.4 mg mL^−1^ for 20 min) showed higher survival percentage and shoots and roots proliferation, although the percentage of explants oxidation was also high (Table 6).

All explants (100%) from the three *C. quitensis* populations, *D. antarctica* and *J. bufonius* that were subcultured on the fourth week post-disinfection developed roots and new shoots, and had no symptoms of yellowness or necrosis 2 months post-subculture (Fig. 1g–k).

## Discussion

According to the results, different species and populations responded differently to the treatments. In terms of contamination percentages, in the Arctowski and Laredo populations of *C. quitensis* (13% in both) and in *D. antarctica* (20%), the lower contamination results were obtained when using calcium hypochlorite as a disinfectant agent (Tables 3–5). The use of this disinfectant has been successful in other studies on different plant species such as *Psidium guajava* L., in which a concentration at 100 mg mL^−1^ used for 15 minutes (equal to the T3 treatment in this work) effectively controlled contamination and reached percentages of around 10%^33^. Meanwhile in *Stevia rebundiana* (Bertoni), contamination percentage was 15% when explant surfaces were adequately disinfected using Ca(ClO)_2_ at 3% (30 mg mL^−1^ for 20 min)^34^. Although sodium hypochlorite is more commonly used for disinfecting explants, calcium hypochlorite has a higher chlorine content, which could explain why the latter is more effective in reducing contamination than the control treatment^35^. Silver nitrate also decreased microbial growth not only for the Arctowski and Laredo populations of *C. quitensis*, but for *D. antarctica* and *J. bufonius* as well. However, this treatment caused high percentages of tissue oxidation in all three species, and in some cases caused explant death. *Trifolium pratense L*. explants showed oxidation within a few hours of being treated with AgNO_3_ at 10 mg mL^−1^ for 1 or 3 min^32^. Similar effects have been observed in *Taxus baccata* L. exposed at 10 mg mL^−1^ concentrations for 3 and 5 min, where 50% and 80% necrosis were recorded, respectively^28^. In the present study, the AgNO_3_ at 10 mg mL^−1^ solution for 10 min (T8) was the treatment that registered the lowest percentage of contamination for *J. bufonius*, and this treatment was more effective than Ca(ClO)_2_ (Table 6). These results are consistent with those obtained in *Prunus cerasus L*. shoots, in which the AgNO_3_ at 10 mg mL^−1^ for 20 min reduced contamination to 3.3%, while the Ca(ClO)_2_ at 50 mg mL^−1^ for 5 min showed 20% contamination^29^. However, in *J. bufonius*, both AgNO_3_ and Ca(ClO)_2_ caused explant death, unlike NaClO (control), which had a survival percentage of 80%. The efficiency of AgNO_3_ as a disinfection solution prior to the establishment of explants *in vitro* may be limited due to the instability of AgNO_3_ in the presence of chemical components that may adhere to or be exuded by the explants or compounds remaining from the processes of washing with tap water, such as chlorides or other salts^36^. Thus, the results indicate that continuing to use the methodology previously established in the Biotechnology and Environmental Studies Laboratory (NaClO at 7.4 mg mL^−1^ for 20 min) is recommended for *J. bufonius*. The silver nanoparticle treatments presented high contamination rates in all populations of *C. quitensis* and in *D. antarctica*, reaching values between 80 and 100%. These results differ from others reported in the literature. For example, in nodal segments of *Valeriana officinalis* L. the microbial contamination after the application of AgNPs at a concentration of 0.1 mg mL^−1^ for 60 min (equal to T9 treatment in this work) resulted in a value of 63%, while the increase in treatment time per 180 min prevented the development of microorganisms in the culture for 89% without causing tissue damage^31^. On the other hand, the application of AgNPs at 0.1 and 0.2 mg mL^−1^ for 7 min decreased in the percentage of contamination in adventitious buds and nodal explants in *Pennisetum alopecuroides*^37^, but caused a 60% reduction in the viability of the shoots on *Prunus amygdalus* x *Prunus persica*^38^. In this work, the reduced number of explants where contamination control was achieved showed a high reduction in their survival or in the proliferation in their tissue (Tables 2–5). The disinfectant properties of AgNPs depend on their shape and size, i.e. bactericidal activity increases as AgNPs size decreases^39^. In strains of *E. coli*, in *Bacillus subtilis* and *Staphylococcus aureus* was determined that the size increase of AgNPs (within a range of 5 to 100 nm) and the concentration increase were needed to inhibit the growth of these bacteria, caused the death up to 99% of them^40^. The size of the AgNPs used as disinfectants in the articles mentioned above were within a range of 10 and 35 nm, which differs from those used in this work (50 nm). This could explain the high percentages of contamination of the explants in the three plant species of the present study. After four weeks post-disinfection treatment, shoot formations were observed in all three species, which was considered to be a good development of the plantlets under *in vitro* conditions. For *C. quitensis*, between 6% and 93% of the plantlets developed shoots, and differences were found between the populations studied. For this species in *in vitro* conditions, without considering the population factor, shoot percentages increasing around 80% have been reported after 4 weeks^15^. In other populations that have previously been studied such as La Parva and La Marisma, the number of shoots was found to be 2.8 and 0.7 on average per plantlet, respectively^16^, which is similar to what was observed in this work (Tables 2–4). In La Marisma, more than 40% of new shoots were observed after 28 days of cultivation^41^. No root development was observed in the Arctowski and Conguillío populations during the evaluation period of this study, but root formation was observed once the plantlets were subcultured. In the Laredo population, roots were developed only in the Ca(ClO)_2_ treatments, which may have been because the explants treated with AgNO_3_ presented oxidation in their tissues, which would have interfered with root formation. The development of flowers in *in vitro* cultures is undesired, as it is considered a symptom of stress^15^. This occurred only in the Arctowski population, where 13% and 46% of plantlets developed flowers, although these values have been close to 10% and 30% in previous studies^13,15^. Shoots and roots developed in all treatments for *D. antarctica*, except for the explants treated with AgNPs, which were almost completely covered by fungi. The number of leaves that developed (between 1 and 5) is similar to what has been reported in *in vitro* plantlets, in which the average number of leaves was around 5^42^. Root development occurred approximately two weeks after treatment which, as reported by Cuba *et al*.^12^, corresponds to a normal period of development for *in vitro* cultures of this species. In *J. bufonius*, the number of leaves produced *in vitro* (ranking between 2.3 and 9.2) was similar, even better, than that observed in plants grown on substrate, which presented an average of 3.5 leaves per plant^11^. This species also showed inflorescence and flower formation, and although this species develops a high number of inflorescences by plants in controlled culture^11^, the micropropagation condition must be improve to avoid this unwanted effect in *in vitro* culture.

## Conclusions

The results obtained for disinfection processes prior to the establishment of plant tissues *in vitro* depended on the different disinfection agents used, the concentrations applied and the time of exposure to the treatment. Results also differed depending on the type of tissue or genotypic variation of the species, which determined the level of tolerance to exposure to each type of disinfectant used, and the capacity of the explant to continue with the micropropagation process^34,43^. In this study, contamination was reduced using calcium hypochlorite and silver nitrate for the Arctowski (pA) and Laredo (pL) populations of *C. quitensis*; however, the latter caused damage through oxidation in the explants, so the use of calcium hypochlorite at 100 mg mL^−1^ for 15 minutes (T3) is recommended for both populations. In pA, although this treatment caused more decreases in the percentage of explant survival and shoot proliferation, it also caused a decrease in the flower appearance percentage. In pL, the same treatment (T3) caused high contamination, but increased the survival and shoot proliferation percentage. Meanwhile, for the Conguillío population (pC) of *C.quitensis*, further studies are recommended to evaluate higher concentrations of calcium hypochlorite and/or increased application times, a combination of treatments or the use of other disinfectants. Treatment 4 (calcium hypochlorite at 100 mg mL^−1^ for 20 minutes) could be used because although deficient, it showed the best results in this work. The use of calcium hypochlorite at 100 mg mL^−1^ 20 minutes (T4) was the most effective for the *D. antartica* species. For *J. bufonius*, continuing to use the protocol previously established by the Biotechnology and Environmental Studies Laboratory (NaClO at 7.4 mg mL^−1^ for 20 min) is suggested, which has been successfully used for the introduction to tissue culture and propagation. Our laboratory has an ongoing development of protocols for both the accelerated propagation and maintenance in minimal growth conditions for short- and medium-term conservation. Thus, these results contribute significantly to the advance in the introduction, propagation and conservation of Antarctic species in the Antarctic Vascular Plant Collection of the Laboratory of Biotechnology and Environmental Studies of the University of Concepción^44^. As of now and according to our knowledge, there is no collection similar to this in any country, so part of this work involves national and international collaboration. Therefore, the protocols developed are of great utility for initiating conservation of these species or others with associated endophytic microorganisms. The conservation and propagation of this genetic resource not only contributes to the development of relevant research for these species, but to efforts to reduce sampling of these species in their natural habitats. This will reduce disturbances in Antarctica, which is still considered a pristine environment.
